# A studyforrest extension, an annotation of spoken language in the German dubbed movie “Forrest Gump” and its audio-description

**DOI:** 10.12688/f1000research.27621.1

**Published:** 2021-01-28

**Authors:** Christian Olaf Häusler, Michael Hanke

**Affiliations:** 1 Institute of Neuroscience and Medicine, Brain & Behaviour (INM-7), Research Center Jülich, Jülich, Nordrhein-Westfalen, 52425, Germany; 2 Institute of Systems Neuroscience, Medical Faculty, Heinrich Heine University, Düsseldorf, Nordrhein-Westfalen, 40225, Germany

**Keywords:** annotation, language, speech, narrative, naturalistic stimulus, fMRI, studyforrest

## Abstract

Here we present an annotation of speech in the audio-visual movie “Forrest Gump” and its audio-description for a visually impaired audience, as an addition to a large public functional brain imaging dataset ( studyforrest.org). The annotation provides information about the exact timing of each of the more than 2500 spoken sentences, 16,000 words (including 202 non-speech vocalizations), 66,000 phonemes, and their corresponding speaker. Additionally, for every word, we provide lemmatization, a simple part-of-speech-tagging (15 grammatical categories), a detailed part-of-speech tagging (43 grammatical categories), syntactic dependencies, and a semantic analysis based on word embedding which represents each word in a 300-dimensional semantic space. To validate the dataset’s quality, we build a model of hemodynamic brain activity based on information drawn from the annotation. Results suggest that the annotation’s content and quality enable independent researchers to create models of brain activity correlating with a variety of linguistic aspects under conditions of near-real-life complexity.

## Introduction

Cognitive and psychiatric neuroimaging are moving towards studying brain functions under conditions of lifelike complexity ^1^^,^^2^. Motion pictures ^3^ and continuous narratives ^4^^,^^5^ are increasingly utilized as so called “naturalistic stimuli”. Naturalistic stimuli are usually designed for commercial purposes and to entertain their audiences. Thus, the temporal structure of their feature space is usually not explicitly known, leading to an “annotation bottleneck” ^6^ when used for neuroscientific research.

Data-driven methods like inter-subject correlation (ISC) ^7^ or independent component analysis (ICA) ^8^ are often used to analyze such fMRI data in order to circumvent this bottleneck. However, use of data-driven methods alone falls short of associating results with particular stimulus events ^9^. Model-driven methods, like the general linear model (GLM), which are based on stimulus annotations can be useful to test hypotheses on specific brain functions under more ecologically valid conditions, to statistically control confounding stimulus features, and to explain not just “how” the brain is responding to a stimulus but also “why” ^10^. Studies using GLMs based on annotations of a stimulus’ temporal structure have elucidated, for example, how the brain responds to visual features of a movie ^11^ or speech-related features of a narrative ^12^. Furthermore, stimulus annotations can inform data-driven methods about a stimulus’ temporal dynamics, or model-driven and data-driven methods can be combined to improve the interpretability of results ^13^.

Here we provide an annotation with exact onset and offset of each sentence, word and phoneme (see Table 1 for an overview) spoken in the audio-visual movie “Forrest Gump” ^14^ and its audio-description (i.e. the movie’s soundtrack with an additional narrator) ^15^. fMRI data of participants watching the audio-visual movie ^16^ and listening to the audio-description ^17^ are the core data of the publicly available *studyforrest* dataset ( studyforrest.org). The current publication enables researchers to model hemodynamic brain responses that correlate with a variety of aspects of spoken language ranging from a speaker’s identity, to phonetics, grammar, syntax, and semantics. This publication extends already available annotations of portrayed emotions ^18^, perceived emotions ^19^, as well as cuts and locations depicted in the movie ^20^. All annotations can be used in any study focusing on aspects of real-life cognition by serving as additional confound measures describing the temporal structure and feature space of the stimuli.

**Table 1.** Overview of the annotation’s content for the audio-description of “Forrest Gump” (i.e. the audio-only variant of the movie) that comprises the additional narrator. Counts are given for the whole stimulus ( all) and its individual segments used during fMRI scanning. The category sentences comprises complete grammatical sentences which are additionally marked in the annotation with a full stop at the end (“my feet hurt.”). It also comprises questions (“do you want a chocolate?”), exclamations (“run away!”), or non-speech vocalizations in quick succession (“ha, ha, ha”), or in isolation (e.g. “Forrest?”, “Forrest!”, “ha”) at time points when speakers switch rapidly. The category words comprises each word or non-speech vocalization (N=202) in isolation.

Category	All	1	2	3	4	5	6	7	8
Sentences	2528	292	366	320	352	344	289	365	200
Words	16187	2089	2162	2115	2035	2217	2033	2322	1214
Phonemes	66611	8802	8727	8770	8557	9197	8353	9351	4854

## Materials and methods

### Stimulus

We annotated speech in the slightly shortened “research cut” ^17^ of the movie “Forrest Gump” and its temporally aligned audio-description ^16^ that was broadcast as an additional audio track for visually impaired listeners on Swiss public television ^15^. The plot of the original movie is already carried by an off-screen voice of the main character Forrest Gump. In the audio-description, an additional male narrator describes essential aspects of the visual scenery when there is no off-screen voice, dialog, or other relevant auditory content.

### Annotation procedure

Preliminary, manual orthographic transcripts of dialogues, non-speech vocalizations (e.g. laughter or groaning) and the script for the audio-description’s narrator were merged and converted to Praat’s ^21^ TextGrid format. This merged transcript contained rough onset and offset timings for small groups of sentences, and was further edited in Praat for manual validation against the actual content of the audio material. The following steps were performed by a single person, already familiar with the stimulus, in several passes to iteratively improve the quality of the data: approximate temporal onsets and offsets were corrected; intervals containing several sentences were split into intervals containing only one sentence; when two or more persons were speaking simultaneously the less dominant voice was dropped; low volume non-speech vocalizations or low volume background speech (especially during music or continuous environmental noise) which were subjectively assessed to be incomprehensible for the audience were also dropped.

We then used the Montreal Forced Aligner v1.0.1 ^22^ to algorithmically identify the exact onset and offset of each word and phoneme. To enable the aligner to look up the phonemes embedded within each word, we chose the accompanying German pronunciation dictionary provided by Prosodylab ^23^ that uses the Prosodylab PhoneSet to describe the pronunciation of phonemes. To improve the detection rate of the automatic alignment, the dictionary was manually updated with German words that occur in the stimuli but were originally missing in the dictionary. The pronunciation of English words and phonemes occurring in the otherwise German audio track was taken from the accompanying English pronunciation dictionary (following the ARPAbet PhoneSet). The audio track of the audio-description was converted from FLAC to WAV via FFmpeg v4.1.4 ^24^ to meet the aligner’s input requirements. This WAV file, the merged transcription, and the updated dictionary were submitted to the aligner that first trained an acoustic model on the data and then performed the alignment.

The resulting timings of words and phonemes were corrected manually and iteratively in several passes using Praat v6.0.22 ^21^: in a first step, onsets and offsets on which the automatic alignment performed moderately were corrected. Some low volume sentences that are spoken in continuously noisy settings (e.g. during battle or hurricane) were removed due to poor overall alignment performance. In a second step, the complete sentences of the orthographic transcription were copied into the annotation created by the aligner. In a third step, a speaker’s identity was added for each sentence (see Table 2 for the most often occurring speakers). During every step previous results were repeatedly checked for errors and further improvements.

**Table 2.** Sentences spoken by the ten most often occurring speakers sorted alphabetically. The narrator only occurs in the audio-description. Overall 97 persons were identified. Names are mostly identical to the names used in ^18^.

Name	All	1	2	3	4	5	6	7	8
Bubba	74	0	16	40	18	0	0	0	0
Forrest	354	22	37	22	48	50	61	49	65
Forrest (child)	19	17	2	0	0	0	0	0	0
Forrest (v.o.)	369	61	48	53	51	37	40	63	16
Hancock	16	16	0	0	0	0	0	0	0
Jenny	177	0	46	30	3	25	0	57	16
Jenny (child)	23	7	16	0	0	0	0	0	0
Lt. Dan	183	0	0	49	33	65	28	0	8
Mrs. Gump	53	38	2	0	0	0	13	0	0
Narrator	903	111	134	78	139	93	115	147	86

We employed the Python package spaCy v2.2.1 ^25^ and its accompanying German language model ( de_core_news_md) that was trained on the TIGER Treebank corpus ^26^ to automatically analyze linguistic features of each word in their corresponding sentence. Non-speech vocalizations were dropped from the sentences before analysis to improve results. We then performed analyses regarding part-of-speech (i.e. grammatical tagging or word-category disambiguation), syntactic dependencies, lemmatization, word embedding (i.e. a multi-dimensional meaning representation of a word), and if the word is one of the most common words of the German language (i.e. if the word is part of a stop list).

### Data legend

The annotation is available in two different versions, both providing the same information: a) as a text-based Praat TextGrid file, and b) as a text-based, tab-separated value (TSV) formatted table. The following descriptions refer to the ten columns of the TSV file, namely onset, duration, person, text, pos, tag, dep, lemma, stop, vector.

**Start** ( start)

The onset of the sentence, word or phoneme. Time stamps are provided in the format seconds.milliseconds from stimulus onset.

**Duration** ( duration)

The duration of the sentence, word or phoneme provided in the format seconds.milliseconds.

**Speaker identity** ( person)

Name of the person that speaks the sentence, word or phoneme. See Table 2 for the ten most often occurring speakers.

**Text** ( text)

The text of a spoken sentence or word, or the pronunciation of a phoneme. Phonemes of German words follow the Prosodylab PhoneSet, English words follow the ARPAbet PhoneSet.

**Simple part-of-speech tag** ( pos)

A simple part-of-speech tagging (grammatical tagging; word-category disambiguation) of words. The tag labels of this simple part-of-speech tagging follow the Universal Dependencies v2 POS tag set ( universaldependencies.org). See Table 3 for a description of the labels and the respective counts of all 15 labels. Nouns that spaCy mistook for proper nouns or vice versa were corrected via script. Additionally in cells of this column, sentences are tagged as SENTENCE, and phonemes are tagged as PHONEME to facilitate filtering in potential further processing steps.

**Table 3.** Simple part-of-speech tagging ( pos) performed by the Python package spaCy ^25^. All 15 labels sorted alphabetically. Descriptions were taken from spaCy.explain(). Non-speech vocalizations ( NONSPEECH) were manually identified. Counts for the whole stimulus ( all) and for each of the eight stimulus segments refer to the audio-description.

Label	Description	All	1	2	3	4	5	6	7	8
ADJ	adjective	916	138	126	106	96	130	118	128	74
ADP	adposition	1429	181	176	176	194	188	183	213	118
ADV	adverb	1332	166	169	220	162	178	169	193	75
AUX	auxiliary	807	102	120	92	96	125	110	112	50
CONJ	conjunction	525	74	63	71	49	61	80	86	41
DET	determiner	1754	257	243	198	219	220	222	254	141
NONSPEECH	non-speech vocalization	202	23	21	9	23	55	44	13	14
NOUN	noun	2620	361	341	332	343	331	356	351	205
NUM	numeral	66	8	11	11	7	4	9	14	2
PART	particle	572	60	100	90	62	83	53	86	38
PRON	pronoun	2348	275	321	328	260	348	262	362	192
PROPN	proper noun	1012	131	135	119	168	162	116	117	64
SCONJ	subordinating conjunction	172	19	18	20	15	31	27	26	16
VERB	verb	2317	285	308	320	319	289	274	349	173
X	other	108	8	10	21	21	11	9	17	11

**Detailed part-of-speech tag** ( tag)

A detailed part-of-speech tagging of words following the TIGER Treebank annotation scheme ^26^ which is based on the Stuttgart-Tübingen-Tagset ^27^. See Table 4 for a description of the labels and the respective counts of the 15 most often occurring labels (overall 43 labels). Nouns that spaCy mistook for proper nouns or vice versa were corrected via script.

**Table 4.** Detailed part-of-speech tagging ( tag) performed by the Python package spaCy ^25^. The 15 most often occurring labels (overall 43 labels) sorted alphabetically. Descriptions were taken from spaCy.explain(). Counts for the whole stimulus ( all) and for each of the eight stimulus segments refer to the audio-description.

Label	Description	All	1	2	3	4	5	6	7	8
ADJA	adjective, attributive	478	73	58	58	51	77	58	70	33
ADJD	adjective, adverbial or predicative	438	65	68	48	45	53	60	58	41
ADV	adverb	1181	146	145	201	143	157	149	174	66
APPR	preposition; circumposition left	1192	156	146	156	152	157	150	178	97
ART	definite or indefinite article	1340	199	183	140	178	159	176	191	114
KON	coordinate conjunction	475	58	58	66	45	58	76	78	36
NE	proper noun	1012	131	135	119	168	162	116	117	64
NN	noun, singular or mass	2620	361	341	332	343	331	356	351	205
PPER	non-reflexive personal pronoun	1638	183	210	221	168	246	176	287	147
PPOSAT	attributive possessive pronoun	274	34	47	36	23	39	32	40	23
PTKVZ	separable verbal particle	353	34	63	49	46	41	33	60	27
VAFIN	finite verb, auxiliary	767	96	108	89	92	116	106	110	50
VVFIN	finite verb, full	1512	181	213	201	202	172	181	228	134
VVINF	infinitive, full	271	37	25	51	32	42	27	40	17
VVPP	perfect participle, full	329	37	40	35	58	44	51	50	14

**Syntactic dependency ( dep)**

Information about a word’s syntactic dependencies with other words within the same sentence. Information follows the TIGER Treebank annotation scheme ^26^ and is given in the format: “arc label;word’s head;word’s child1, word’s child2, ...”, where the “arc label” (see Table 5) describes the type of syntactic relation that connects a ”child” (the current word) to its “head”.

**Table 5.** Syntactic dependencies ( dep) performed by the Python package spaCy ^25^. The 15 most often occurring labels (overall 37 labels) sorted alphabetically. Descriptions were taken from spaCy.explain(). Counts for the whole stimulus ( all) and for each of the eight stimulus segments refer to the audio-description.

Label	Description	All	1	2	3	4	5	6	7	8
cd	coordinating conjunction	335	48	44	48	34	41	53	42	25
cj	conjunct	524	65	74	88	53	65	80	65	34
cp	complementizer	160	17	17	20	16	29	25	21	15
da	dative	170	15	30	27	19	23	18	27	11
ju	junctor	130	10	13	16	12	16	22	31	10
mnr	postnominal modifier	245	30	29	33	44	31	28	27	23
mo	modifier	2634	349	345	355	327	356	334	384	184
nk	noun kernel element	3763	516	482	448	475	507	485	551	299
oa	accusative object	1036	117	139	149	148	146	126	134	77
oc	clausal object	732	98	86	97	94	105	97	115	40
pd	predicate	301	39	50	40	25	45	41	38	23
pnc	proper noun component	154	36	19	15	14	28	22	15	5
ROOT	root of sentence	2417	285	349	322	336	317	267	358	183
sb	subject	2231	280	306	271	276	301	281	340	176
svp	separable verb prefix	355	36	65	45	49	43	33	56	28

**Lemmatization** ( lemma)

The base form (root) of a word.

**Common Word** ( stop)

This column’s cell provides information if the word is part of a stop list, hence one of the most common words in the German language or not ( True vs. False).

**Word embedding** ( vector)

A 300-dimensional word vector providing a multi-dimensional meaning representation of a word. Out-of-vocabulary words with a vector consisting of 300 dimensions of zeroes were set to # to save space.

### Dataset content

The annotation comes in two different versions. First, as a text-based TextGrid file ( annotation/ fg_rscut_ad_ger_speech_tagged.TextGrid) to be conveniently edited using the software Praat ^21^. Second, as a text-based, tab-separated-value (TSV) formatted table ( annotation/fg_rscut_ ad_ger_speech_tagged.tsv) in accordance with the brain imaging data structure ( BIDS) ^28^. The dataset and validation data are available from Open Science Framework, DataLad and Zenodo (see Underlying data) ^29^^,^^30^^,^^31^. The source code for all descriptive statistics included in this paper is available in code/descriptive-statistics.py (Python script).

## Dataset validation

In order to assess the annotation’s quality, we investigated if contrasting speech-related events to events without speech lead to increased activation in areas known to be involved in language processing ^32^. Moreover, we tested if two similar linguistic concepts (proper nouns and nouns) providing high semantic information contrasted with a concept providing low semantic information (coordinate conjunctions) lead to increased activation in congruent brain areas.

We used a dataset providing blood oxygenation level-dependent (BOLD) functional magnetic resonance imaging (fMRI) data of 20 subjects (age 21–38 years, mean age 26.6 years, 12 male) listening to the 2 h audio-description (7 Tesla, 2 s repetition time, 3599 volumes, 36 axial slices, thickness 1.4 mm, 1.4 × 1.4 mm in-plane resolution, 224 mm field-of-view) ^17^. Data were already corrected for motion at the scanner computer. Further, individual BOLD time-series were already aligned by non-linear warping to a study-specific T2*-weighted echo planar imaging (EPI) group template (cf. ^17^ for exact details).

All further steps for the current analysis were carried out using FEAT v6.00 (FMRI Expert Analysis Tool) ^33^ as part of FSL v5.0.9 (FMRIB’s Software Library) ^34^. Data of one participant were dropped to due to invalid distortion correction during scanning. Data were temporally high-pass filtered (cut-off 150 s), spatially smoothed (Gaussian kernel; 4.0 mm FWHM), and the brain was extracted from surrounding tissue. A grand-mean intensity normalization of the entire 4D dataset was performed by a single multiplicative factor.

**Figure 1.**
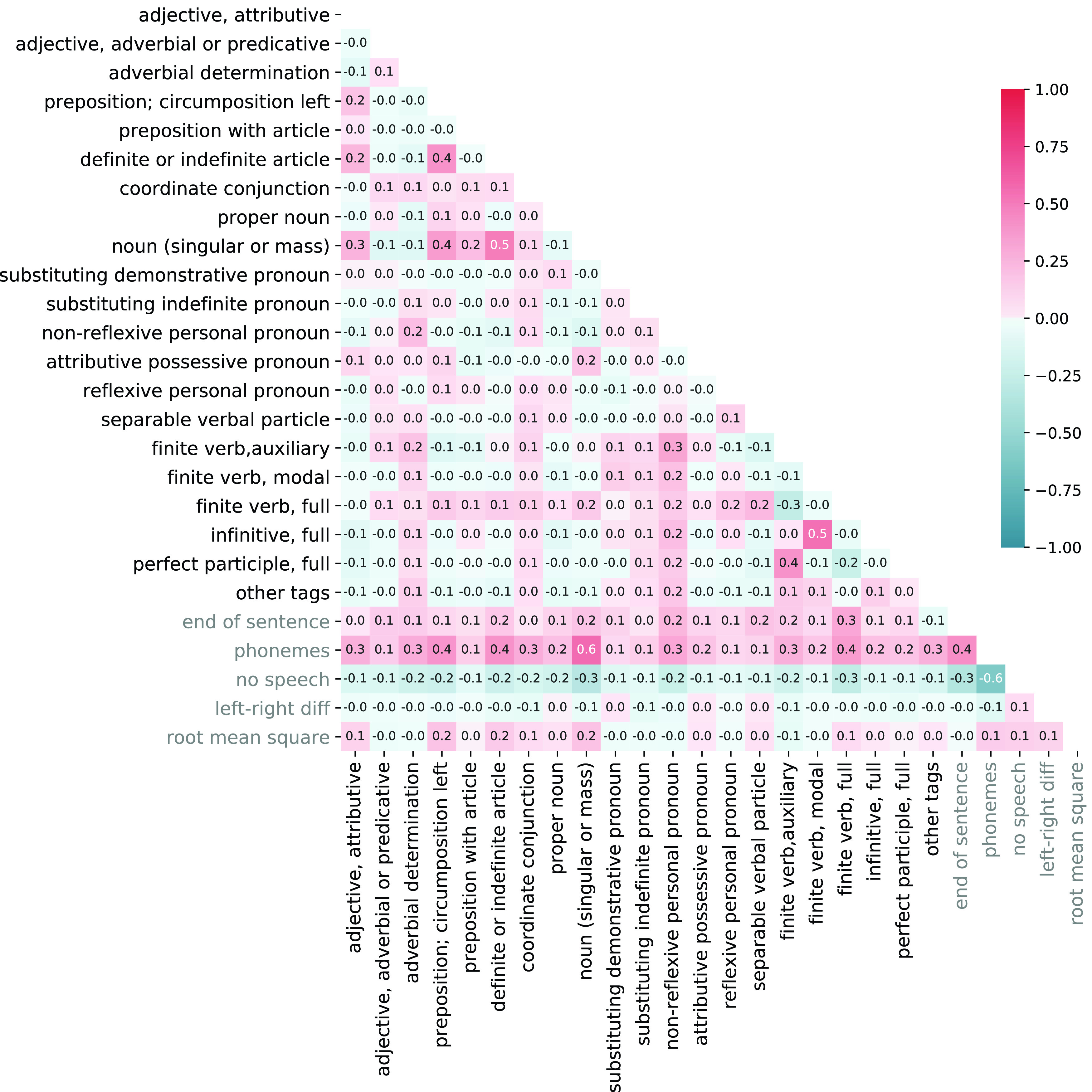
Pearson correlation coefficients of the 26 regressors used in the analysis to validate the annotation. Regressors were created by convolving the events with FSL’s “Double-Gamma HRF” as a model of the hemodynamic response function, temporally filtered with the same high-pass filter (cut-off 150 s) as the BOLD time series, and concatenated across runs before computing the correlation.

We implemented a standard three-level, voxel-wise general linear model (GLM) to average parameter estimates across the eight stimulus segments, and later across 19 subjects. At the first level analyzing each segment for each subject individually, we created 26 regressors (see Table 6) based on events drawn from the annotation. The 20 most often occurring detailed part-of-speech labels ( nn with N=2620 to prf with N=157) were modeled as boxcar function from onset to offset of each word. The remaining other part-of-speech labels were pooled to a single new label ( tag_other; N=1123) and modeled as a boxcar function from a word’s onset to offset. The 80 most often occurring phonemes ( n with N=6053 to IY1 with N=32) were pooled to phonemes (N=65251) and modeled as boxcar function from a phoneme’s onset to offset. The end of each complete grammatical sentence was modeled as an impulse event (N=1651) to capture variance correlating with sentence comprehension. “No-speech” events ( no-sp; N=264) serving as a control condition were created such that a sufficient number of events and a minimum separation of speech and non-speech events were achieved. Events were randomly positioned in intervals without audible speech that lasted at least 3.6 s. Each event of the no-speech condition had to have a minimum distance of 1.8 s to any onset or offset of a word, and to any onset of another no-speech event. A length of 70 ms was chosen for no-speech events matching the average length of phonemes. Lastly, we used continuous bins of information about low-level auditory features (left-right difference in volume and root mean square energy) that was averaged across the length of every movie frame (40 ms) to capture variance correlating with assumed low-level perceptual processes. Time series of events were convolved with FSL’s “Double-Gamma HRF” as a model of the hemodynamic response function to create the actual regressors. The Pearson correlation coefficients of the 26 regressors across the time course of all stimulus segments can be seen in Figure 1. Temporal derivatives were also included in the design matrix to compensate for regional differences between modeled and actual HRF. Finally, six motion parameters were used as additional nuisance regressors and the design was subjected to the same temporal filtering as the BOLD time series. The following three *t*-contrasts were defined: 1) words (all 21 tag-related regressors) > no-speech ( no-sp), 2) proper nouns ( ne) > coordinate conjunctions ( kon), and 3) nouns ( nn) > coordinate conjunctions ( kon).

**Table 6.** Overview of events that were used to create the 26 regressors of the GLM analysis. The respective counts are given for the whole stimulus and the eight segments that were used during fMRI scanning. The 20 most often occurring labels from the detailed part-of speech tagging ( tag) were used as such. Words belonging to all other labels were pooled to tag_other. The label sentence contains the end of complete grammatical sentences. The label phones contains events of the 80 most often occurring phonemes (phoneme n with N=6053 to phoneme IY1 with N=32). The label no-sp represents moments when no speech was audible. fg_ad_lrdiff (left-right volume difference) and fg_ad_rms (root mean square energy) were compute for and averaged across every movie frame (40 ms) via Python script. Events were convolved with FSL’s “Double-Gamma HRF” to create the regressors. The correlation of these regressors over the time course of the whole stimulus can be seen in Figure 1.

Label	Description	All	1	2	3	4	5	6	7	8
adja	adjective, attributive	478	73	58	58	51	77	58	70	33
adjd	adjective, adverbial or predicative	438	65	68	48	45	53	60	58	41
adv	adverb	1181	146	145	201	143	157	149	174	66
appr	preposition; circumposition left	1192	156	146	156	152	157	150	178	97
apprart	preposition with article	233	24	28	20	42	31	32	35	21
art	definite or indefinite article	1340	199	183	140	178	159	176	191	114
kon	coordinate conjunction	475	58	58	66	45	58	76	78	36
ne	proper noun	1012	131	135	119	168	162	116	117	64
nn	noun, singular or mass	2620	361	341	332	343	331	356	351	205
pds	substituting demonstrative pronoun	192	16	32	31	25	33	27	17	11
pis	substituting indefinite pronoun	217	36	30	35	28	30	21	23	14
pper	non-reflexive personal pronoun	1638	183	210	221	168	246	176	287	147
pposat	attributive possessive pronoun	274	34	47	36	23	39	32	40	23
prf	reflexive personal pronoun	157	18	25	15	23	17	26	19	14
ptkvz	separable verbal particle	353	34	63	49	46	41	33	60	27
vafin	finite verb, auxiliary	767	96	108	89	92	116	106	110	50
vmfin	finite verb, modal	183	28	21	29	24	28	15	30	8
vvfin	finite verb, full	1512	181	213	201	202	172	181	228	134
vvinf	infinitive, full	271	37	25	51	32	42	27	40	17
vvpp	perfect participle, full	329	37	40	35	58	44	51	50	14
tag_other	all other TAG categories	1123	153	165	174	124	169	121	153	64
sentence	complete grammatical sentences	1651	205	231	200	215	212	198	249	141
phones	80 most often occurring phonemes	65251	8589	8534	8597	8387	8976	8184	9232	4752
no-sp	no-speech	264	16	20	23	50	25	27	56	47
fg_ad_lrdiff	left-right volume difference	180133	22574	22075	21925	24425	23125	21975	27175	16859
fg_ad_rms	root mean square	180133	22574	22075	21925	24425	23125	21975	27175	16859

The second-level analysis that averaged contrast estimates across the eight stimulus segments per subject was carried out using a fixed effects model by forcing the random effects variance to zero in FLAME (FMRIB’s Local Analysis of Mixed Effects) ^35^^,^^36^. The third level analysis which averaged contrast estimates across subjects was carried out using a mixed-effects model (FLAME stage 1) with automatic outlier deweighting ^36^^,^^37^. Z (Gaussianised T/F) statistic images were thresholded using clusters determined by Z>3.4 and a corrected cluster significance threshold of p<.05 ^37^. Brain regions associated with observed clusters were labeled using the Jülich Histological Atlas ^38^^,^^39^ and the Harvard-Oxford Cortical Atlas ^40^ provided by FSL.

**Figure 2.**
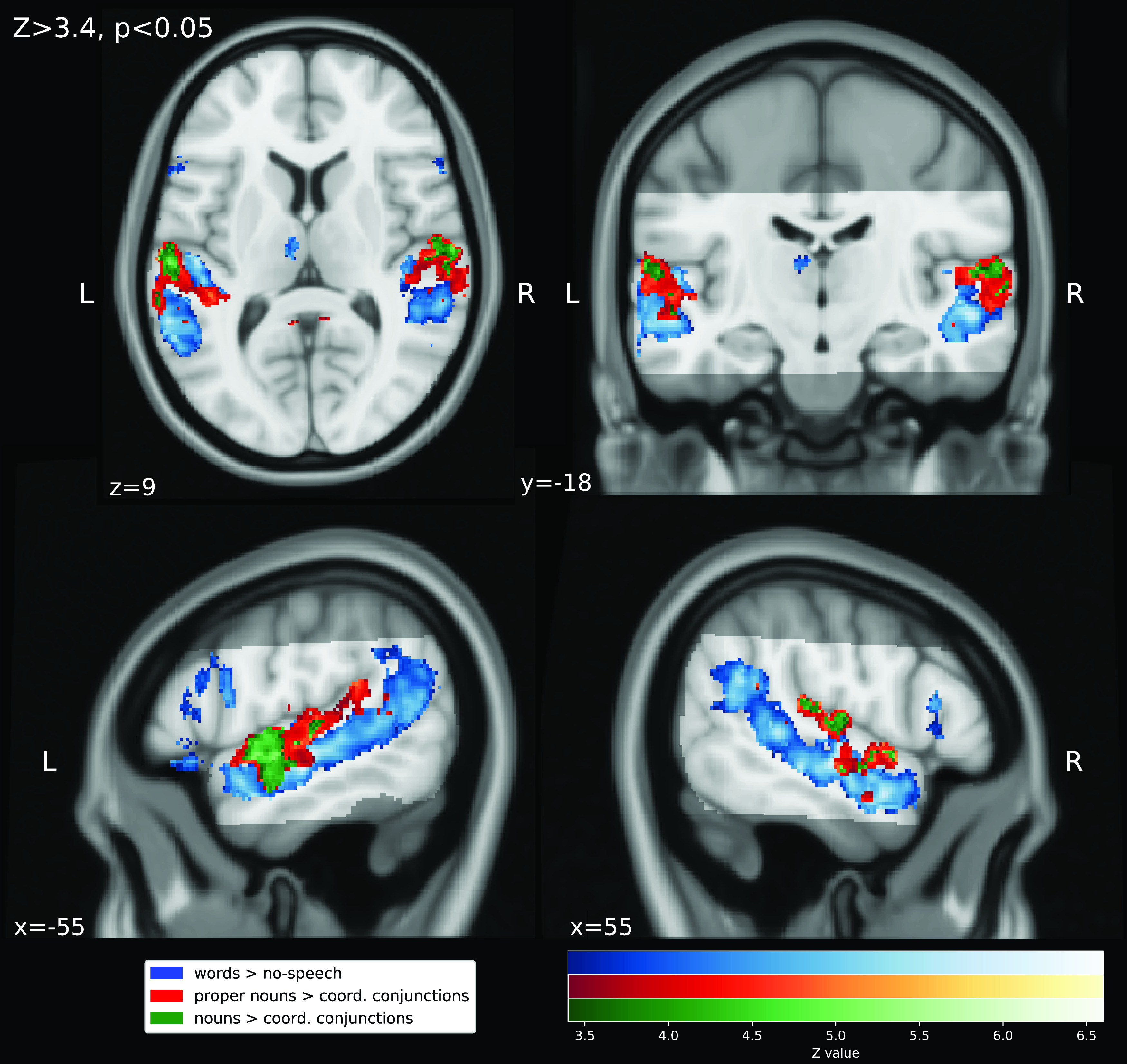
Results of the mixed-effects group-level (N=14) GLM *t*-contrasts for the audio-description of the movie “Forrest Gump”. Significant clusters (Z>3.4, p<0.05 cluster-corrected) are overlaid on the MNI152 T1-weighted head template (grey). Light grey: the audio-description dataset’s field-of-view (cf. ^17^).

Figure 2 depicts the results of the three contrasts (z-threshold Z>3.4; p<.05 cluster-corrected). The contrast words > no-speech yielded four significant clusters (see Table 7): one left-lateralized cluster spanning from the angular gyrus and inferior posterior supramarginal gyrus across the superior and middle temporal gyrus, including parts of Heschl’s gyrus and planum temporale. A second left cluster in (inferior) frontal regions, including precentral gyrus, pars opercularis (Brodmann Areal 44; BA44) and pars triangularis (BA45). Similarly in the right hemisphere, one cluster spanning from the angular gyrus across the superior and middle temporal gyrus but including frontal inferior regions (pars opercularis and pars triangularis). A fourth significant cluster is located in the left thalamus.

**Table 7.** Significant clusters (z-threshold Z>3.4; p<.05 cluster-corrected) for the contrast words (all 21 tag-related regressors) > no-speech. Clusters sorted by voxel size. The first brain structure given contains the voxel with the maximum Z-Value, followed by brain structures from posterior to anterior, and partially covered areas (l. = left; r. = right; c. = cortex; g. = gyrus).

			Max location (MNI)			Center of gravity (MNI)			
Voxels	*p*_*corr.*_	Z-max	x	y	z	x	y	z	Structure
14990	<.001	6.31	-49	-24.7	6.35	-54.8	-32.5	3.73	l. Heschl’s g.; lateral superior occipital c., angular g., superior & middle temporal g. (posterior to anterior); parts of supramarginal g. & planum temporale
14469	<.001	6.48	55	-14.9	-6.9	54.1	-23.1	0.374	r. superior temporal g.; angular g., superior (and middle) temporal g. (posterior to anterior), Heschl’s g.; parts of supramarginal g., planum temporale, pars opercularis (BA44) & pars triangularis (BA45)
1971	<.001	5.26	-51.1	25.6	-10.5	-53.6	17.8	10.2	l. frontal orbital c.; pars opercularis (BA44), pars triangularis (BA45); parts of precentral g.
217	.002	4.55	-4.48	-13.7	10.3	-6.46	-14.9	9.96	l. thalamus

The contrast proper nouns > coordinate conjunctions yielded nine significant clusters (see Table 8): one left-lateralized cluster spanning from the angular gyrus across planum temporale and superior temporal gyrus, partially covering the Heschl’s gyrus, into the anterior middle temporal gyrus. A largely congruent but smaller cluster in the right hemisphere. Two clusters in posterior cingulate cortex and precuneus of both hemispheres. Three small clusters in the right occipital pole, right Heschl’s gyrus and left superior lateral occipital pole.

**Table 8.** Significant clusters (z-threshold Z>3.4; p<.05 cluster-corrected) for the contrast proper nouns ( ne) > coordinate conjunctions ( kon). Clusters sorted by voxel size. The first brain structure given contains the voxel with the maximum Z-Value, followed by brain structures from posterior to anterior, and partially covered areas (l. = left; r. = right; c. = cortex; g. = gyrus).

			Max location (MNI)			Center of gravity (MNI)			
Voxels	*p*_*corr.*_	Z-max	x	y	z	x	y	z	Structure
7691	<.001	6.23	-61.2	-22.3	11.6	-55.9	-20.7	4.03	l. planum temporale; posterior inferior supramarginal g., superior temporal g., planum polare, parts of posterior angular g., Heschl’s g., middle temporal gyrus
5928	<.001	5.5	57.5	-26.2	15.9	58.2	-15.8	3.55	r. planum temporale; Heschl’s g., superior temporal g., planum polare, temporal pole; parts of angular g. & posterior inferior supramarginal gyrus
479	<.001	4.62	-5.42	-32.3	25.3	-4.28	-39.4	22.8	l. posterior cingulate g.
420	<.001	4.85	-4.76	-71.4	40.1	-3.74	-68.5	36.2	l. precuneus
407	<.001	5.07	6.83	-40.1	24.5	6.67	-38.7	23.1	r. posterior cingulate c.
294	<.001	4.57	17	-69.1	34.6	17.7	-67.1	34.9	r. precuneus
121	.024	3.95	8.12	-98.2	0.359	8.75	-97.7	-3.15	r. occipital pole
117	.027	4.38	36.9	-24.8	4.55	37.4	-23	3.09	r. Heschl’s g.
115	.029	4.08	-44.6	-71.7	21.7	-43.6	-70.8	23.4	l. superior lateral occipital c.

The contrast nouns > coordinate conjunctions yielded four significant clusters (see Table 9): two clusters that are slightly smaller than the lateral temporal clusters of contrast nouns > coordinate conjunction. In this case, spanning from angular gyrus in the left hemisphere and from planum temporale in the right hemisphere into the anterior part of superior temporal cortex. Finally, two small right-lateralized clusters in the right posterior cingulate gyrus and right precuneus.

**Table 9.** Significant clusters (z-threshold Z>3.4; p<.05 cluster-corrected) for the contrast nouns ( nn) > coordinate conjunctions ( kon). Clusters sorted by voxel size. The first brain structure given contains the voxel with the maximum Z-Value, followed by brain structures from posterior to anterior, and partially covered areas (l. = left; r. = right; c. = cortex; g. = gyrus).

			Max location (MNI)			Center of gravity (MNI)			
Voxels	*p*_*corr.*_	Z-max	x	y	z	x	y	z	Structure
3166	<.001	5.75	-61.3	-10.6	-2.93	-57.7	-14.3	1.47	l. anterior superior (and middle) temporal g.; planum temporale, planum polare, anterior superior temporal g.; part of posterior supramarginal g., Heschl’s g.
1753	<.001	4.99	63.3	-15.1	8.41	58	-13	4.02	r. planum temporale, anterior superior temporal g., planum polare; part of& part of Heschl’s G.
166	.004	4.5	6.83	-40.1	24.5	7.01	-39.7	24.2	r. posterior cingulate g.
149	.008	4.13	18.2	-67.8	36	19.8	-66.4	34.6	r. precuneus

For the contrast words > no-speech, results show increased hemodynamic activity in a bilateral cortical network including temporal, parietal and frontal regions related to processing spoken language ^32^^,^^41^^,^^42^. These clusters resemble results of previous studies that implemented an ISC approach to analyze fMRI data of naturalistic auditory stimuli ^5^^,^^43^^,^^44^. We do not find significantly increased activations in midline areas (like the posterior cingulate cortex and precuneus or anterior cingulate cortex and medial frontal cortex) which showed synchronized activity across subjects in previous studies. In this regard, our results are similar to ^4^ who implemented both an ISC and a GLM analysis. In this study, the ISC analysis showed synchronized activity in midline areas but the GLM analysis contrasting blocks of listening to narratives to blocks of a resting condition showed significantly decreased activity in these areas.

The two contrasts that contrasted nouns and proper nouns respectively to coordinate junctions yielded increased activation partially located in early sensory regions (Heschl’s Gyrus; ^45^) and most prominently adjacent regions bilaterally (planum temporale; superior temporal gyrus; ^46^^,^^47^). We chose nouns and proper nouns for these two contrasts because they represent linguistically similar concepts but are uncorrelated in the German language and stimulus (cf. Figure 1). We contrasted nouns and proper nouns respectively to coordinate conjunctions because nouns and proper nouns are linguistically different to coordinate conjunctions as well as uncorrelated. Despite the fact that nouns and proper nouns are uncorrelated, both contrasts lead to largely spatially congruent clusters. Results suggest that models based on our annotation of similar linguistic concepts correlate with hemodynamic activity in spatially similar areas. We confirmed the validity of these interpretation by testing if the spatial congruency could be attributed to a negative correlation of coordinate conjunctions with the modeled time series which turned out not to be the case. In summary, results of our exploratory analyses suggest that the annotation of speech meets basic quality requirements to be a basis for model-based analyses that investigate language perception under more ecologically valid conditions.

## Data availability

### Underlying data

Zenodo: A studyforrest extension, an annotation of spoken language in the German dubbed movie “Forrest Gump” and its audio-description (annotation). https://doi.org/10.5281/zenodo.4382143 ^29^.

Dataset 1. The annotation (v1.0; registered) as a tab-separated-value (TSV) formatted table and a text-based TextGrid file (the native format of the software Praat).

Zenodo: A studyforrest extension, an annotation of spoken language in the German dubbed movie “Forrest Gump” and its audio-description (validation analysis). https://doi.org/10.5281/zenodo.4382188 ^30^.

Dataset 2. The data of the analysis (v1.0; registered) that we ran as a validation of the annotation’s content and quality.

Open Science Framework: studyforrest-paper-speechannotation. https://doi.org/10.17605/OSF.IO/GFRME ^31^.

The paper as LATE X document, and accompanying datasets 1 and 2 (up-to-date; unregistered) accessible as DataLad (RRID:SCR_003931) datasets.

Data are available under the terms of the Creative Commons Attribution 4.0 International license (CC-BY 4.0).

### Author contributions

COH designed, performed, and validated the annotation, and wrote the manuscript. MH provided critical feedback on the procedure and wrote the manuscript.
