# Supplementary material for: A studyforrest extension, an annotation of spoken language in the German dubbed movie “Forrest Gump” and its audio-description
Source: F1000Res. 2021 Jan 28;10:54. [Version 1] doi: 10.12688/f1000research.27621.1 (PMC7921887; doi:10.12688/f1000research.27621.1)
Supplement: Peer review history

# Peer review: Reviewer response for version 1

**DOI:** 10.5256/f1000research.30529.r78808

**Published:** 2021-03-01

**Authors:** Roberta Rocca

**Affiliations:** 1 Psychoinformatics Lab, Department of Psychology, The University of Texas at Austin, Austin, TX, USA

The authors introduce a new set of linguistic annotations for the *studyforrest *dataset, an open naturalistic fMRI dataset where subjects are presented audio-visual or audio-only versions of the movie Forrest Gump. This new corpus of annotations is an extremely valuable addition to the dataset. The availability of high-quality annotations of linguistic content enables researchers to easily set up and conduct analyses tapping into the neural correlates of phonetic, semantic and syntactic processing. Furthermore, making multi-level (word-, phoneme-, and sentence-level) time-stamped transcripts of the stimulus makes it possible to extract any linguistic features beyond those directly provided by the authors. Access to the new annotations is made simple through DataLad. Additionally, the authors report the results of a few validation analyses where annotated features are used in a GLM setting to retrieve established effects (e.g., neural correlates of speech and semantic processing), which speaks in favor of their reliability.

 Overall, the manuscript is clear, the annotation pipelines used are solid and the data sharing format is easy to navigate. It is a great contribution to the field, and to open science culture more generally. I have a few suggestions that mostly concern: a) increasing the overall clarity and readability of the manuscript; b) making it easier for readers who are not familiar with the dataset to understand how annotations map onto the various parts of the studyforrest dataset and use them; c) making it easier for readers with limited expertise in linguistic annotations to track the meaning of individual features.

**Introduction**

1. In the introduction, the authors make a general point about model-driven GLM analyses providing a series of advantages over data-driven methods, especially in terms of interpretability. They write that “use of data-driven methods alone falls short of associating results with particular stimulus events”, while GLMs “allow to test hypotheses on specific brain functions under more ecologically valid conditions, to statistically control for confounding stimulus features, and to explain not just “how” the brain is responding to a stimulus, but also why”. A few objections can be moved to these claims (or to the way they are presented here). While GLMs are, on the surface, more easily interpretable, their use (especially in naturalistic contexts) poses a few challenges. For example, a) global characteristics of the stimulus make interpretation of baselines tricky (and their commensurability across datasets challenging), with consequences on the interpretation of the effects of specific features; b) the choice of covariates, while allowing to control for collinearity, can also alter the interpretation of the effects of individual features – in other words, interpretation is conditional on the model; c) causal claims (aka the *why*) are notoriously hard to make on the basis of linear models alone. While I am sympathetic to the idea that a lot can be gained by the use of these simple tools, authors could consider nuancing some or further specifying some of their claims.
2. My next (related) point concerns the link between the annotations presented by the authors and GLMs. In the introduction, the authors seem to present the usability of their annotations as almost constrained to analytic contexts where GLMs are used or as heuristics tools for data-drive methods. But nothing prevents these annotations from being used as either predictor or target features in other analysis frameworks (e.g., predictive models). The authors could consider being a bit more ambitious, and slightly reframing the first paragraphs along the lines of these considerations.
3. In the introduction, the authors claim that “stimulus annotations can inform data-driven methods about a stimulus’ temporal dynamics”. I think the gist of it is clear, but maybe a more concrete example or wording would help.
4. A minor point about wording, should “the current publication” be “the present publication” or “this publication”? Not a native speaker, so it may just be an issue with my English (in which case, please ignore this comment).

**Materials and methods**

1. It may be beneficial to mention early in the paper that the movie is presented in German. It is also a great feature of the dataset, being it one of the very few dataset – to my knowledge – with stimuli *not *in English, which is quite crucial for matters of cross-linguistic generalization.
2. One aspect that is not entirely clear from the paper is which component of the *studyforrest* dataset the annotations refer to / can be used for. Can they only be used for data from subjects where the movie was only presented auditorily, or can the same annotations (filtering out “narrator” rows) also be used for those parts of the dataset where participants are presented with the movie both auditorily and visually? I think it will be highly beneficial to the manuscript to a) provide a little recap of what studyforrest is, its sub-components, and where to find them; b) make it more explicit for which batches of subjects/tasks these new annotations can be used.
3. More of a clarification question than a suggestion: the onsets in the annotations tsv file refer to the full movie file. It should be possible to cross-reference these onsets with run- and subject-specific events files from the BIDS dataset. If so, could more details on how to get from onsets in the annotation files to run-specific GLM-ready onsets be provided (at a high level, e.g., “onsets in the annotation files can be cross-referenced with time-stamps in the event files x and x to retrieve subject-specific and run-specific onsets …”)? Disclaimer: I am relatively new to BIDS, and this may be a trivial point (in which case ignore this comment).
4. Once again a minor point, but the fact that annotations are BIDS-compliant and shared in tsv could already be mentioned in the introduction (or even in the abstract).
5. In “Annotation procedure”, the authors introduce the fact that the stimulus is also annotated at the sentence-level. However, a definition of a sentence is only given in the description for Table 1. Could a quick reference of what counts as a sentence be added to the main text?
6. The authors mention having dropped the “less dominant” voice when speakers overlap. Could a couple of words be added on how that was defined (volume? character prominence?)? And since some could argue these events are also potentially interesting bits of information, do instances of overlaps represent a significant portion of the stimulus, or does this occur only in few instances?
7. On page 4, the authors talk about feature extraction in terms of analysis (e.g., “automatically analyze linguistic features of each word in their corresponding sentence” and performed “analyses” regarding part of speech). Probably a matter of taste, but would rather refer to these steps as “extraction” of linguistic features; to me, the use of “analysis” suggests that more has been performed on the features (e.g., visualization or some form of validation) than mere feature extraction.
8. Which type of word embeddings were extracted should be specified.
9. SpaCy has very good documentation explaining the interpretation of each feature and how pipelines for extraction work (e.g. https://spacy.io/usage/linguistic-features). References to that (e.g., simply links to the documentation) could be added, for example in the sentence “We then performed analyses regarding part-of-speech… “ whenever each feature/pipeline is introduced. It would make it easier for non-linguists to get a feel for what features mean, and for experts to dig into the details of the specific pipelines used.
10. The paragraph “Annotation procedure” may benefit from adding an example, e.g., a sample sentence from the transcript with the corresponding annotation. It would help readers (especially those who do not have deep expertise in linguistics) to visualize what features are about and visualize the different levels of annotation. I understand this may be tricky to do without disrupting the flow of the text though and will leave it to the authors to decide whether to implement it or not.
11. In the caption for table 2, can the expression “sentences spoken by the ten most often occurring speakers” be streamlined?
12. Can a sample of the resulting annotation dataset (whose components are described in “data legend”) be displayed as exemplification, e.g., just the head of the table or something similar?

**Dataset validation**

1. Can the (run-level) design matrix be shown, possibly with labels for the regressors? Would make for a nice visual, and help readers better understand the structure of the analysis.
2. It is not entirely clear to me why authors annotated for the onset/duration of phonemes, but only using the 80 most frequent phonemes. What is the role of that regressor in the model, what is it meant to code and/or control for? Can this be motivated more in detail in the text?
3. Another aspect of the analysis which is not entirely clear to me is why “no speech” events were created ad hoc. Wouldn’t the group-level z-map for a binary regressor coding for the onset of all speech events (or even just all words) do the same job without the need for mock events? Or couldn’t a comparison between word events and events tagged as NONSPEECH in the ‘pos’ column of the dataset be an easier implementation of the same question? The analyses are convincing and sound to me considering the purpose of this paper, so I’m not necessarily suggesting to change them. But the authors could consider further clarifying the rationale for their choices.
4. When mentioning annotations of low-level auditory features, the authors refer to “continuous bins of information about low-level feature”. The wording here could be made more transparent.
5. Can the authors provide some motivation for the choice of thresholding at z>3.4?
6. I find the representation of contrasts in Figure 2 a bit confusing, because of the combinations of high overlaps between effects and of the colormap (not a lot of contrast between the three colors). If that is not uniquely my concern, the authors could consider either splitting the map into separate figures or experimenting with alternative color combinations.

It was a pleasure to review this work. Great set of resources, and great contribution to the field!

Are sufficient details of methods and materials provided to allow replication by others?

Yes

Is the rationale for creating the dataset(s) clearly described?

Yes

Are the datasets clearly presented in a useable and accessible format?

Yes

Are the protocols appropriate and is the work technically sound?

Yes

Reviewer Expertise:

naturalistic fMRI, natural language processing, psychoinformatics

I confirm that I have read this submission and believe that I have an appropriate level of expertise to confirm that it is of an acceptable scientific standard, however I have significant reservations, as outlined above.

# Peer review: Reviewer response for version 1

**DOI:** 10.5256/f1000research.30529.r78810

**Published:** 2021-02-16

**Authors:** Martin Wegrzyn

**Affiliations:** 1 Department of Psychology, Bielefeld University, Bielefeld, Germany

The authors present a word-for-word annotation of an audio version of the movie “Forrest Gump”, as used in the “studyforrest” project, for which 7T fMRT data of 20 participants is available (as described in other publications). The presented work provides a number of syntactic and semantic labels, which can be useful for studying language using this naturalistic stimulus. An additional validation of the dataset, by using a subset of its parameters as a design for fMRI analysis, illustrates its applicability. The rationale for creating the dataset is clearly described. The protocols used are appropriate and the work is technically sound. Overall, sufficient details of methods and materials are provided for the main syntactic markers of speech. However, some of the additional semantic annotations are not described in enough detail, in my opinion. The created datasets are clearly presented and are in a usable and accessible format, especially if one is a Python user.

 This is a high-quality work that I think will substantially boost the usability of the 2014 “studyforrest” publication by Hanke and colleagues. I think naturalistic stimuli are very valuable; Especially for data sharing, their openness to different scientific questions is a strong asset. Right now (2021), many PhD-students will have a hard time collecting fMRI data or are unable to collect data altogether. I would not be surprised if this publication (together with Hanke 2014 Scientific Reports) will give rise to a dozen or more valuable publications on language-fMRI.

 Having worked with some “studyforrest” data myself, and having tried to annotate the stimulus, I think I can appreciate the level of dedication required to complete an annotation at the level of the present one (I know I couldn't do it). Because a naturalistic stimulus is not designed for experimental usage, many parts of it will be more ambiguous than one would like it to be. Stemming in part from my own experience, I therefore also have a number of specific questions regarding how the authors solved those challenges.

 Questions and comments:

- At the end of the first paragraph/beginning of the second, the authors write: *“* *Thus, the temporal structure of their feature space is usually not explicitly known, leading to an* *'* *annotation bottleneck* *'[6]* *when used for neuroscientific research. Data-driven methods like inter-subject correlation (ISC)* *[7]* ^**^ *or independent component analysis (ICA)* *[8]* ^**^ *are often used to analyze such fMRI data in order to circumvent this bottleneck. However, use of data-driven methods alone falls short of associating results with particular stimulus event* *s [9]”*
  I understand the point the authors are making. Namely, that it usually takes much more time to annotate the data than to collect them. However, I would like to suggest that the wording (“temporal structure”, “feature space”, “bottleneck”), especially in the first paragraph of the article, might be a bit too challenging to immediately understand. Maybe a gentler introduction to the topic would be helpful for some readers.
  Also, I think that the point the authors make is not 100% valid: The study by Hasson et al. (cited in [7]) actually circumvented the problem of annotating the whole stimulus, by using “reverse correlation”: That is, only interpreting the time points where activity is highest. This allowed Hasson and colleagues to interpret effects on a substantive level, just by showing the frames of the movie for which brain activity was highest. I think this is also a useful and economical approach – but certainly more limited than a full annotation.
- In the description of Table 1 the authors write: *“The category 'sentences' comprises complete grammatical sentences which are additionally marked in the annotation with a full stop at the end (* *'* *my feet hurt.* *'* *). It also comprises questions (* *'* *do you want a chocolate?* *'* *), exclamations (* *'* *run away!* *'* *), or non-speech vocalizations in quick succession (* *'* *ha, ha, ha* *'* *), or in isolation (e.g.* *'* *Forrest?* *'* *,* *'* *Forrest!* *'* *,* *'* *ha* *'* *) at time points when speakers switch rapidly. The category words comprises each word or non-speech vocalization (N=202).”*
  I completely understand that the additional coding of whether a speech episode is a legal sentence in German would go beyond the scope of the present work. Also, users will be able to code this themselves, according to their needs, using the detailed tagging provided by the authors (and the dot at the end is also useful, though not a perfect marker (e.g. “lief und lief.” or “Hörfilm e. V.”)). However, I think that calling a variable or feature “sentences”, when it really is not coding the presence of a sentence, could be a source of future errors. The same goes for “words”. So maybe the authors would want to consider using different labels for those two features.
- Related to the point above, I think that sometimes a single sentence has longer pauses and is then divided into multiple “sentences”. Take the narration at the very end of the movie: *“S* *ie wird wieder angehoben, ...* *” , “* *und schwebt auf die Kamera zu.* *”* is coded as two sentences. Researchers interested in syntax might benefit from being aware that they have some additional work to do (which is only fair!), in order to merge those speech episodes which are really a single sentence. Additionally, if they define the sentences differently, they will have to do a different tagging of the words, too. I do not suggest that the authors need to do any additional work, but I just want to point out that the label “sentences” could mislead some language researchers.
  Apart from sentences being divided by pauses, there is the question of punctuation. Given that the stimulus is audio only, my personal experience is that it is sometimes difficult to decide on a definitive punctuation (where commas should occur or if a comma or a full stop should be used). I think that in German commas play a bigger role in defining the syntactic structure of a sentence than they might do e.g. in English. Therefore, I would suggest that some brief discussion of the general limitations of deriving syntactic information from an audio stimulus might be a useful addition to the article (of course only if the authors agree with my premise).
- “Data Legend” > “Common Word (stop)”: The source or the definition of the stop list would be helpful for users who would want to cite it in their publications
- “Data Legend” > “Word embedding”: This is a very rich and useful resource and I was immediately able to do some interesting analyses using the 300-dimensional word vector. For example, correlating the values of all words with each other gave some very interesting and plausible results (e.g. color words clustering together etc.). I think this itself could merit a separate publication with a focus on semantics. As it currently stands, there is however no information about how these data were generated and how future users could cite them and find out more about them. Therefore, I think that a bit more information could significantly increase the impact of this resource.
- Also, when playing around with it, I noticed that sometimes two different words have the exact same 300-dimensional vector. For example:
  strahlend-bewölkt
  überqueren -durchqueren
  braun-olivgrün
  gepackten-schlamm
  schütteln-wischen
  Is this correct behavior and why would two different words have the exact same values? Especially for a pair like “gepackten”-”Schlamm” that might be surprising.
- “Dataset validation”: maybe some brief examples for the “coordinating conjunction” and different “nouns” could be given for illustrative purposes
- “Dataset validation”: why were non-speech events not modeled with a boxcar, spanning their whole duration, when this was otherwise possible for single words. It would seem to me that the duration of individual words would make a boxcar function much more problematic than using a boxcar for speech-free periods (which are often short, but there are some really long ones in there as well, which might not be fully utilized when modeled with a default length of 70ms – which would amount to a stick function).
- “Dataset validation”: how do the non-speech events correlate with the other features (i.e. could they be included in the heatmap in Fig 1?)
- Tables 7-9: could it be better to round the values to full mm? I would argue that even for 7T data (smoothed with 4mm FWHM), sub-mm precision for the MNI-coordinates might imply an accuracy that is not obtainable
- Figure 2 legend (and pages 7, 12): I recently learned that “cf.” is used to point to contrasting information (for example, if there is literature making an opposing point; https://blog.apastyle.org/apastyle/2010/05/its-all-latin-to-me.html).

Thank you!

Are sufficient details of methods and materials provided to allow replication by others?

Partly

Is the rationale for creating the dataset(s) clearly described?

Yes

Are the datasets clearly presented in a useable and accessible format?

Yes

Are the protocols appropriate and is the work technically sound?

Yes

Reviewer Expertise:

fMRI, language

I confirm that I have read this submission and believe that I have an appropriate level of expertise to confirm that it is of an acceptable scientific standard, however I have significant reservations, as outlined above.

# Peer review: Reviewer response for version 1

**DOI:** 10.5256/f1000research.30529.r78806

**Published:** 2021-02-16

**Authors:** Giada Lettieri

**Affiliations:** 1 Social and Affective Neuroscience Group, MoMiLab, IMT School for Advanced Studies Lucca, Lucca, Italy

The current Data Note reports and describe an extensive dataset including speech annotation in the audio-visual movie "Forrest Gump". To assess the consistency of annotation, the brain hemodynamic activity elicited by the contrast of speech events versus no speech was also investigated.

 The dataset here provided is richly detailed and is a great addition to the work already done in the studyforrest project. The significant effort carried out by the authors is particularly relevant for data sharing and for future investigations on language and lifelike experiences.

 In light of all this, I would only have two suggestions:

1. I think it would help readers that are not familiar with the original dataset to have in the header of tables the indication "run 1, run 2..." or "segment 1, segment 2...", instead of just the numbers from 1 to 8.
2. It was not fully clear to me which kind of algorithm the authors used to obtain the word-embedding. As different algorithms provide different results for word embedding, I think it would be interesting to specify which one of these was employed in their analyses (e.g., GloVe, word2vec).

In conclusion, this extension enriches the already available annotations of the original neuroimaging dataset and concur in building a comprehensive and valuable description of a naturalistic stimulation.

Are sufficient details of methods and materials provided to allow replication by others?

Yes

Is the rationale for creating the dataset(s) clearly described?

Yes

Are the datasets clearly presented in a useable and accessible format?

Yes

Are the protocols appropriate and is the work technically sound?

Yes

Reviewer Expertise:

Neuroscience

I confirm that I have read this submission and believe that I have an appropriate level of expertise to confirm that it is of an acceptable scientific standard.
